# Whole genome surveys of rice, maize and sorghum reveal multiple horizontal transfers of the LTR-retrotransposon *Route66* in *Poaceae*

**DOI:** 10.1186/1471-2148-9-58

**Published:** 2009-03-16

**Authors:** Anne Roulin, Benoit Piegu, Philippe M Fortune, François Sabot, Angélique D'Hont, Domenica Manicacci, Olivier Panaud

**Affiliations:** 1 Laboratoire Génome et Développement des Plantes, UMR CNRS/IRD/UPVD, Université de Perpignan, 52, avenue Paul Alduy, 66860 Perpignan, cedex, France; 2 Centre de Coopération International en Recherche Agronomique pour le Developpement (CIRAD), UMR1096, Avenue Agropolis, 34398 Montpellier, Cedex 5, France; 3 UMR de Génétique Végétale, INRA UPS INA-PG CNRS, Ferme du Moulon, 91190 Gif-sur-Yvette, France

## Abstract

**Background:**

Horizontal transfers (HTs) refer to the transmission of genetic material between phylogenetically distant species. Although most of the cases of HTs described so far concern genes, there is increasing evidence that some involve transposable elements (TEs) in Eukaryotes. The availability of the full genome sequence of two cereal species, (*i.e*. rice and *Sorghum*), as well as the partial genome sequence of maize, provides the opportunity to carry out genome-wide searches for TE-HTs in *Poaceae*.

**Results:**

We have identified an LTR-retrotransposon, that we named *Route66*, with more than 95% sequence identity between rice and *Sorghum*. Using a combination of *in silico *and molecular approaches, we are able to present a substantial phylogenetic evidence that *Route66 *has been transferred horizontally between Panicoideae and several species of the genus *Oryza*. In addition, we show that it has remained active after these transfers.

**Conclusion:**

This study constitutes a new case of HTs for an LTR-retrotransposon and we strongly believe that this mechanism could play a major role in the life cycle of transposable elements. We therefore propose to integrate classe I elements into the previous model of transposable element evolution through horizontal transfers.

## Background

Horizontal Transfers (HTs) are defined as the transmission of genetic information between reproductively isolated species. Contrasting with vertical transmission (*i.e*. from parents to their progeny), this process has for a long time been considered as an unusual phenomenon. However, many studies have shown that HTs are frequent among prokaryotes and that they widely contribute to lineage evolution by acquisition of new genes [1,2]. Several recent reports have shown that gene flow can also occur between reproductively isolated eukaryotic species through HTs. These can involve genes [3-6] as well as transposable elements [7-9].

Transposable elements (TEs) are mobile DNA sequences which have the capacity to move from one location to another in their host genome. They are divided into two classes according to their mode of transposition (see [10], for the most recent review). Class I, or retrotransposons, transpose through a "copy and paste" mechanism. After their transcription, the RNA is reverse transcribed and integrated into the genome, leading to the duplication of the original copy. Retrotransposons can in some cases rapidly increase their copy number. In plants, it has been shown that these elements, notably the Long Terminal Repeat (LTR)-retrotransposons, are the main cause of genome size increase in the genus *Oryza *[11], in cotton [12] or in maize [13], beside polyploidy. Class II elements, or transposons, transpose through a "cut and paste" mechanism. These elements are excised and reintegrated elsewhere in the genome.

Because TEs from both class I and II can be found as free molecules (either RNA or DNA) during at least one step of the transposition cycle, they are considered to be more prone to HTs than other genomic sequences. In animals, many studies have shown that TEs can move between distantly related species, as for the genus *Drosophila *[14] where the *P-element *invasion constitutes one of the best described cases of HT in eukaryotes [7,15,16]. In plants, only two studies have described TE HTs, the first concerning the transfer of a *Mu-like *element (transposon) between foxtail millet (*Setaria italica*) and rice (*Oryza sativa*) [8] and the second involving multiple HT of a retrotransposon between seven species of the genus *Oryza *[9].

The scarcity of cases of TE HTs in plants may be an indication that they are rare phenomena. Alternatively, it could only reflect the technical difficulties raised by their detection and correct characterization. In that case, one could anticipate that the recent accumulation of genomic resources through large scale genome sequencing projects will provide more opportunities to study HTs in eukaryotes. As an example, a large amount of genomic sequences is now available for cereals such as rice, *Sorghum *and maize. Since rice diverged from maize and *Sorghum *50–70 Mya, it excludes the transfer of genetic information by hybridization between these species and makes them appropriate to study HTs following a comparative genomic approach. We previously demonstrated the multiple HT of a retrotransposon within the genus *Oryza *[9]. By combining genome-wide comparative genomic analyses and molecular approaches, we present phylogenetic evidence of the HT of an LTR-retrotransposon, *Route66*, from a sugarcane relative to several rice species. Based on the accumulation of evidence of the occurrence of TE-HTs in eukaryotes, we also propose to include LTR-RTs in a general model of TE-mediated plant genome evolution.

## Results

### *Route66 *characterization

**Figure 1:**
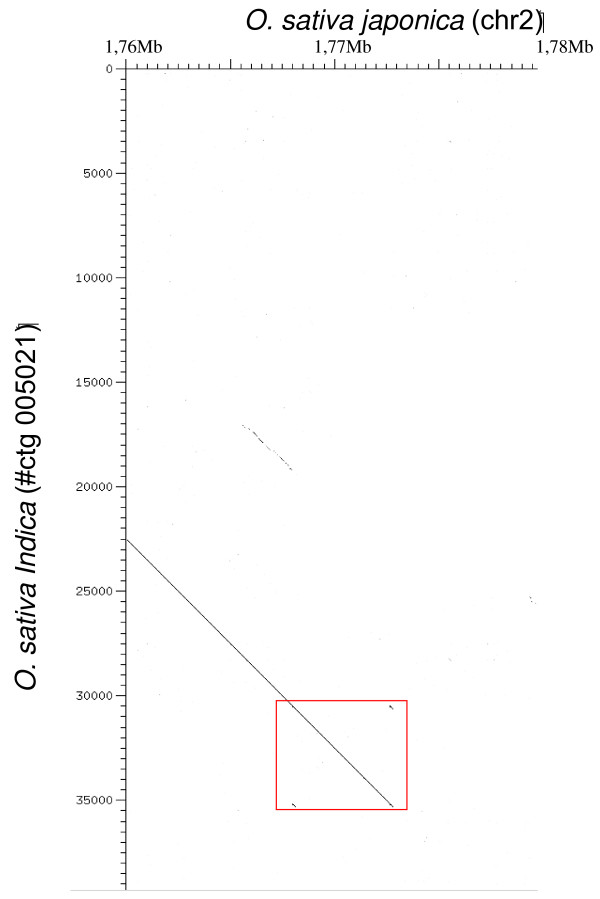
**Dot plot analysis**. Horizontal: *O. sativa *ssp. *japonica*. Vertical: *O. sativa *ssp. *indica*. The red square corresponds to *Route66 *sequence. Left border of *Route66 *is highly conserved between *indica *and *japonica*.

In a previous study [17], we identified *Route66*, an LTR-retrotransposon which is found in two copies in the genome of the cultivated rice species, *Oryza sativa *ssp. *Japonica*. One is located on chromosome 2 (nt 1 767 933 to nt 1 772 818, referred to hereafter as Osj2) and the other on chromosome 6 (nt 25 706 265 to nt 25 701 456, referred to hereafter as Osj1). *Route66 *is a 4,890 bp long LTR-retrotransposon with short 203 bp LTRs. In addition, only one copy of *Route66 *is found in the genome of the *indica*-type variety 93–11. The copy of *Route66 *of *Oryza sativa *ssp. *japonica *located on chromosome 2 is 99.5% identical to that of *indica*. Dot plot analyses (Figure 1) show that the *japonica *and *indica *sub-species share the insertion of *Route66 *on chromosome 2, which implies that either this insertion predates the radiation of the two subspecies or that it was introgressed from one subspecies into the other.

**Figure 2:**
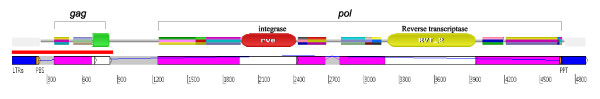
**Rice *Route66 *complete element using Artemis and Pfam annotation**. The *gag *and *pol *regions are shown. The red line represents the the 1 kb cloned fragment in the different species.

The structural annotation of *Route66 *confirmed that it is a complete element in rice, *i.e*. that it harbors all the features required for its mobility: LTRs, PBS, PPT and a complete *gag-pol *Open Reading Frame (Figure 2). In addition, the sequence identity observed between both copies of *O. sativa *ssp. *Japonica *is 99.4% (Table 1). Using a substitution rate of 1,3 × 10^-8 ^mutation/site/year [18], we estimated that *Route66 *has been active in rice in the last 230,000 years.

**Table 1:** *Route66 *sequence identity and Ka/Ks values.

*Route66*	identity (%)	KaKs
osj1/osj2	99.4	0.94
osj1/osi	99.1	1
osj2/osi	99.5	0.56
osj1/sb1	95.4	0.46
osj1/zm4	92.4	0.18
zm4/sb1	91.1	0.25

Osj: *O. sativa *ssp. *japonica*; Osi: *O. sativa *ssp. *indica *; zm: *Zea mays*; sb: *Sorghum bicolor*.

### *Route66 *as a new candidate for Horizontal Transfers

#### *In silico *detection

**Figure 3:**
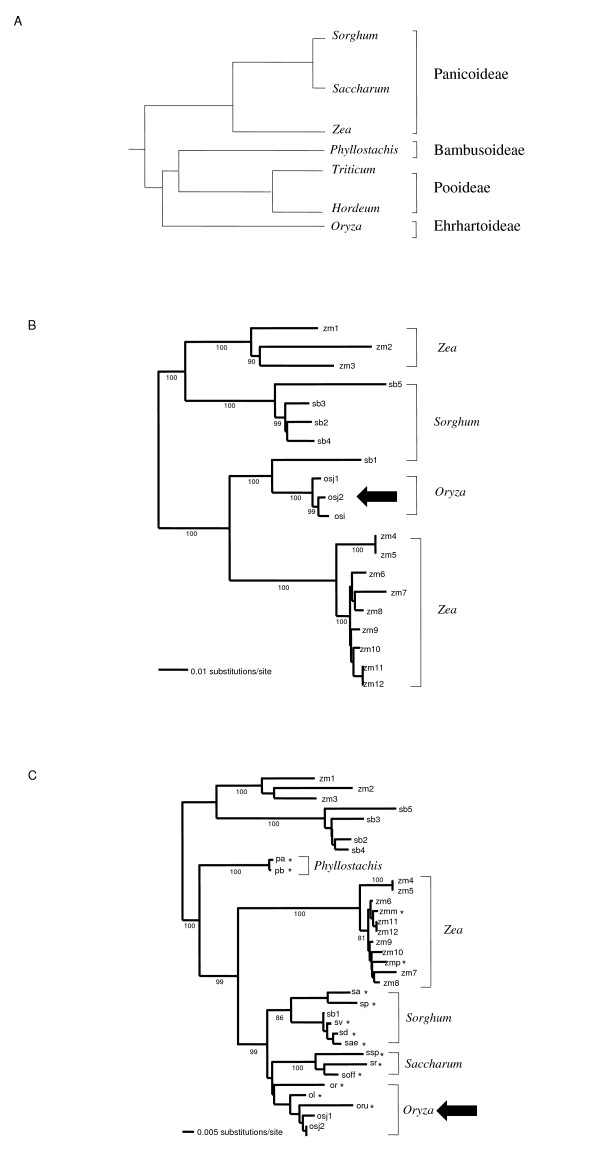
**Phylogenetic analysis**. A) Simplified species phylogeny adapted from [21] B) *Route66 *relationships between maize, *Sorghum *and rice. The tree was drawn using complete copies of *Route66*. C) *Route66 *relationship between maize, *Sorghum*, rice, sugarcane and their wild relatives and bamboo, based on the alignment of the 1 kb fragment. * represents sequences obtained by PCR cloning. Arrow shows sequences responsible for the phylogenetic incongruence. Numbers indicate bootstrap values in percent. Only the values higher than 80% were kept. Species abbreviations: zm: *zea mays *ssp. *mays*;zmm: *zea mays *ssp *mexicana*; zmp: *zea mays *ssp *parviglumis; *sb: *Sorghum bicolor*; sa: *Sorghum arundinaceum*; sp: *Soghum propinquum*; sd: *Sorghum drummondii*; sae: *Sorghum aethiopicuum; *sv: *Sorghum virgatum*. pa: *Phyllostachis aurea*; pb: *Phyllostachis bisetii*; sr: *Saccharum robustum*; soff: *Saccharum officinarum*; ssp: *Saccharum spontaneum*; osj:*Oryza sativa japonica*; osi: *Oryza sativa indica*; or: *Oryza ridleyi*; ol: *Oryza longistaminata*; oru: *Oryza rufipogon*.

In order to investigate the possible origin and the evolution of *Route66 *in grasses, we looked for its presence in either the maize or the *Sorghum *genomes. We mined 12 additional complete copies of the element from maize and 5 from *Sorghum*. For each species, the copies of *Route66 *exhibit a high sequence identity, suggesting that they have been active recently in these genomes (Additional file 1). Surprisingly, the rice *Route66 *copies display more than 95% sequence identity with those found in *Sorghum bicolor*, which is unexpected given the date of their radiation, *i.e*. more than 50 Mya [19]. Moreover, this value is higher than that observed between the maize and *Sorghum *copies of *Route66 *(i.e. 91% on average), although these two species diverged only 12 Mya [20](see Table 1 and Additional files 1 and 2 for detailed results and sequence alignments). We also compared these values with those for seven orthologous genes in rice, maize and *Sorghum *(Table 2). On average, these genes are 85% identical, which indicates that the *Route66 *LTR-retrotransposon is far more conserved between rice and *Sorghum *than these seven genes. We also studied the phylogenetic relationships between all the *Route66 *elements found in rice, *Sorghum *and maize genomes. The phenetic tree (Figure 3B) clearly shows that the rice copies form a cluster with that of *Sorghum*. However, this result is incongruent with the species phylogenetic relationships established with known genes [21](Figure 3A).

**Table 2:** Interspecific sequence identities and Ka/Ks values for seven genes; size indicates the number of bp used for the computation.

	Identity (%)			KaKs			size (bp)
	rice/maize	rice/sorghum	sorghum/maize	rice/maize	rice/sorghum	sorghum/maize	
waxy	84.9	86	94.3	0.133	0.131	0.086	638
nod	89.4	89.1	95.1	0.247	0.196	0.044	900
gid	83.8	84.4	94.1	0.28	0.247	0.226	1.068
AK070134	81.9	85.6	90.7	0.036	0.052	0.023	636
AK072921	86.4	87.5	96.8	0.177	0.173	0.122	1.500
AB055156	80.1	82.5	95.1	0.023	0.035	0.017	732
AK072088	74.6	81.9	83.8	0.096	0.174	0.037	1.332

#### Wet lab validation

In order to confirm the phylogenetic incongruence revealed by the *Route66 *sequences of rice, *Sorghum *and maize, we PCR amplified, cloned and sequenced 1 kb fragments of *Route66 *from various species of *Oryza*, *Sorghum *and *Zea*. We completed this analysis with the tentative cloning of *Route66 *homologs in bamboo, *Saccharum*, millets, wheat and *Brachypodium*. We then obtained additional sequences for three wild rice, three *Saccharum*, two bamboos, two teosintes and five wild *Sorghum *species. For each of the accessions of wild rice, *Saccharum *and *Sorghum bicolor *used in the study, we PCR amplified and cloned the *Adh1 *gene and compared the sequences obtained with that deposited in Genbank for the corresponding species. We therefore ruled out the possibility that our results were due to DNA contamination or mislabelling.

The phenetic tree obtained from the cloned sequences is given in figure 3C. This tree displays some phylogenetic incongruences, as in the case of that based on the *in silico *data above. Both *Oryza *and *Phyllostachis *genus (bamboo) sequences are embedded among Panicoideae whereas, according to the phylogeny of grasses, they should appear as outgroups (Figure 3A). All homologs cloned from *Oryza *species (*O. sativa, O. rufipogon, O. longistaminata *and *O. ridleyi*) form a clear cluster near the sugarcane accessions. Interestingly, *O. sativa *sequences show a 4.5% divergence with that of the wild sugarcane, *Saccharum officinarum *(see Additional file 3 for sequence identities). By using a substitution rate of 1,3 × 10^-8 ^mutation/site/year [18], we estimate that this corresponds to a 1,5 My old radiation whereas both species diverged more than 50 Mya. Surprisingly, *Phyllostachis *sequences are only 9% divergent from the copies of maize and *Sorghum *orthologs which correspond to a 3,3 MY radiation whereas Bamboo and Panicoideae diverged 50 MYa.

The subsequent cloning of *Route66 *in these additional species provided a better understanding of the origin of the HT. If the transfer was recent, it is expected that the copies of both the donor and the receiver species should exhibit a high sequence identity. As a consequence, they should appear in the same cluster in the phylogenetic tree. *Route66 *is 95.5% identical between rice and *Saccharum officinarum*, however the sequences of the genus *Oryza *are clustered near to, although not within, those of *Saccharum*. Further studies should be made to identify the species at the origin of the transfer. Nevertheless, we expect that this species should be phylogenetically close (or belong) to the *Saccharum *genus. A similar observation is made for the transfer involving *Phyllostachis *species. A larger sample of Poaceae taxa should be tested to infer more precisely the origin of these transfers.

### Comparison of the evolutionary dynamics of *Route66 *with that of selected genes in rice, *Sorghum *and maize

For the seven genes mentioned above, we performed an interspecific non-synonymous to synonymous substitution ratio (Ka/Ks) analysis by comparing maize, *Sorghum *and rice orthologs. Table 2 shows that all the genes studied are submitted to purifying selection. All Ka/Ks ratio are lower than 0,3 (Table 2) and they all display similar interspecific sequence identities. We performed a similar analysis on *Route66 *and we observed that this element is under selective constraints when interspecific comparisons are performed (Table 1 and Additional file 4).

## Discussion

### Evidence for horizontal transfer of *Route66*

Transposable elements are known to evolve faster than genes and are rapidly eliminated through unequal and illegitimate recombination [22-24]. Since rice and *Sorghum *diverged some 50–70 Mya [19,25,26], homologous retrotransposons are not expected to be found among these species or, at least, they should exhibit a very low sequence identity. On one hand, *Route66 *displays a high sequence identity (>95%) between rice and *Sorghum *throughout the element despite their radiation date (*i.e*. at least 50 MYa). On the other hand, *Route66 *is more conserved between these species than between *Sorghum *and maize (>91% whereas these two genera only diverged 12 MYa). This constitutes our first argument in favour of HT. Furthermore, *Route66 *is also more conserved between rice and *Sorghum *than genes which are under selective pressure (*i.e*. 85% sequence identity on average) and this constitutes the second and even stronger argument in favour of the HT.

However, two other mechanisms, *i.e*. strong selective pressure throughout the sequence of this element and the reduction of mutation rate in the region flanking the insertions could alternatively lead to the conservation of *Route66 *between rice, *Sorghum *and sugarcane.

TE domestication, defined as the co-option of TE-encoding proteins into functional host protein is one of the process which could be responsible for selective pressure on a TE sequence [27]. A number of studies have demonstrated that domestication is common in some animal genomes [27] and most of them involved a transposase-encoding sequence [28,29]. Through Ka/Ks analyses, we showed that *Route66 *has been subjected to purifying selection, which could be in accordance with a putative functional role of this element. However, this could also reflect the fact that non-functional TEs can not transpose and are rapidly eliminated from the genome through deletions, therefore inducing a bias in the Ka/Ks ratio. However, in the case of domestication, although the element evolves slowly, the topology of the phenetic tree obtained with *Route66 *should be similar to that obtained with the genes classically used for this purpose such as the *waxy *or *phytochrome *genes. This is clearly not the case as shown in Figures 3A, B and 3C since all sequences from the *Oryza *genus (genomic sequences as well as 1 kb fragments) are clustered with those of the Panicoideae (maize, *Sorghum *and sugarcane). They clearly cluster near the sugarcane sequences whereas, according to the phylogenetic relationship of these species, the *Oryza *genus is obviously out of the Panicoideae sub-family. This phylogenetic incongruence is thus in agreement with the sequence identities that were calculated. The high nucleotide sequence identity between rice and *Sorghum *concerns the whole element. No case of domestication of a complete element (including the non-coding LTR regions) has ever been demonstrated so far. For these reasons, we rule out the possibility of the domestication of the TEs.

An alternative explanation is that the elements could be inserted into a region with a reduced mutation rate. Our Ka/Ks survey for the genes flanking both insertions in *O. sativa *ssp. *japonica *showed that they are submitted to selective pressure but exhibit sequence identity similar to that of randomly chosen genes such as *Waxy, Nod2 *and *Gid1 *(Table 2). We therefore conclude that both insertion regions of *Route66 *in rice are not under a stronger selection than other parts of the genome.

Considering all these results and taking into account current knowledge on transposable element evolution, we propose that *Route66 *has been horizontally transferred into the rice genome. Moreover, we used sugarcane to estimate the age of the transfer. Sugarcane and rice copies harbour 95.5% identity (Additional file 3) therefore we estimate that the HT occurred recently, during the last million years. However, both sub-species, *indica *and *japonica*, share a common insertion on chromosome 2 (therefore inherited from their common ancestor, see Figure 1). Knowing that divergence between *indica *and *japonica *occurred during the last million years [30], these results tend to demonstrate that the transfer of *Route66 *occurred just before or concomitantly with the subspecies radiation. Moreover, the genome of *O. sativa *ssp *japonica *harbours one more copy than that of *O. sativa *ssp. *indica*. This indicates that *Route66 *has been active since the sub-speciation.

### Evidence of multiple horizontal transfers

The sequences obtained for the wild rice species, *O. rufipogon*, *O. longistaminata *and *O. ridleyi*, are clustered with that of *O. sativa*. The wild rice copies are more than 98.5% identical to that of *O. sativa*, which is incongruent with the species radiation since, for example, *O. sativa *and *O. ridleyi *diverged 25 Mya. This data is therefore in agreement with the occurrence of a horizontal inheritance of *Route66 *in the genus *Oryza*. However, further studies should be made to test if the HT occured independently from the donor species to the four *Oryza *species or if the copy was first transferred to one rice species and then spread through the genus *Oryza*. Nevertheless, it was previously demonstrated that the LTR-retrotransposon *RIRE1 *has been horizontally transferred between seven wild rice species [9]. We therefore consider that *Route66 *could constitute a second example of multiple HTs in the genus *Oryza*.

We suspect that another HT of *Route66 *occured between a Panicoideae and *Phyllostachis *species. *Phyllostachis *sequences display more than 92% sequence identity with some sequence of maize and *Sorghum*. Moreover, *Phyllostachis *sequences are included in the cluster formed by the *Route66 *homologs from the Panicoideae species, although these sequences should be clearly separated according to the species radiation (Figure 3A). The hypothesis of HT in *Phyllostachis *is reinforced by the fact that *Route66 *was not found in the Triticeae. Even if we can not exclude that this absence is due to to a PCR bias, one explanation is that *Route66 *has not been vertically inherited in grasses although we can not rule out that the element was lost in the ancestor of the Poideae sub-family. This could be tested by the analysis of a larger sample of Pooideae species. The high conservation of *Route66 *between *Phyllostachis *and maize as well as the phylogenetic incongruences are unexpected and could be the result of a second HT of *Route66 *involving Bambusoideae. However, this needs to be ascertained by supplementary analyses.

### Mechanisms involved in Horizontal Transfers

Based on what is known in both animals and plants, three main mechanisms have been invoked to explain HTs [31]. The first is a direct plant-to-plant transfer. This has been well described in the case of gene transfer between parasitic angiosperms and their hosts [3,32,33]. Direct plant-to-plant transfer was estimated to be responsible for 26 HTs of plant mitochondrial genes to *Amborella *[3]. Even if no case of direct plant-to-plant HTs has been reported yet for TEs, this process is likely to occur for any DNA sequence (either genes or TEs). In our case, no rice parasitic plants are known but we cannot exclude the possibility that such plants existed in the past and could have been at the origin of HTs.

An alternative hypothesis is transfer by hybridization and introgression. This hypothesis remains attractive since interspecific hybridization is a common process in plants, presumed to be at the origin of most allopolyploidization events. Many examples of introgressions have been described and are considered to be responsible for organellar gene transfers in plants [34]. However, this mechanism is only possible if the species are close enough to hybridize spontaneously, which is not the case for the Ehrhartoideae (rice) and Panicoideae.

Vector mediated transfers constitutes a third possible mechanism. This could explain HTs between distant species and many studies suggest that bacteria [35] or fungi [6] could be responsible for HTs. Only one example clearly demonstrated the transfer from a reptile to a virus, which constitutes a first step to a vector-mediated transfer [36]. One could suppose that the integration of a retroelement into a viral genome (or other vectors such as insects) could occur in the same way in plants. This could explain why, if several organisms share common parasites, some retrotransposons could be involved in multiple HTs. This still needs to be tested by further experiments to fully understand the mechanisms involved in HTs.

### Transpositional activity of *Route66 *and potential impact on genome evolution

The two *Route66 *copies of *O. sativa *ssp. *japonica *are 99.4% identical. This indicates that *Route66 *has been active recently, *i.e*. during the last 230,000 years. In plants, few LTR retrotransposons are known to be still active. These are for example *Tos17 *in rice [37], *Tnt1 *in tobacco [38] or *BARE1 *in barley [39], whereas most TEs are silenced either by the methylation and/or the small RNA pathways [40,41]. As an example, the *CAC1 *element is transpositionaly activated when present in a *ddm1 *hypomethylation background in *Arabidopsis *[42]. Hirochika et al. have also demonstrated that *Tto1*, a tobacco TE, can transpose in a heterologous systems such as rice or *Arabidopsis thaliana *even if the copy number is positively correlated with DNA methylation, leading to rapid silencing of the element [43]. One could therefore propose that by invading a new genome a transferred element could escape epigenetic silencing and be active after its transfer. This could explain why 3,000 and 300 copies of the horizontally transferred copy of *RIRE1 *are now present in the *O. minuta *and *O. granulata *genomes respectively [9]. The activation process could favor the retention of the transferred copy in the new genome by decreasing the probability of its elimination by genetic drift [44] and could explain why several cases of TE-HTs have been described. In our case, *Route66 *has not undergone any burst of transposition because it is only present in two copies in the rice genome. However the transpositional activity of the transferred copies of *Route66 *possibly explains how it could have spread in several taxa of the grass family. More generally, the transpositional activity of transposable elements after their HTs could explain their evolutive success in eukaryotes lineages since it allows retrotransposons to persist after invasion of a new genome [45].

## Conclusion

There is no doubt that TE HTs could play a role in genome and thus in species evolution by generating variability [46], since TEs are known to have an impact on genes [27,47] and on genome dynamics [11,24,48]. In addition, there is some evidence that horizontally transferred TEs could lead to structural and functional modifications in the recipient species. As an example, the *P-element *is responsible for genetic abnormalities such as high mutation rate, chromosome breakage or sterility in *Drosophila melanogaster *when crossing females lacking *P-element *and males harbouring more than 30 copies of this element [49,50]. Moreover, HTs often involve active elements (as for the *P-element, RIRE1 *or *Route66*) which could make them responsible for modification in both genome structure and gene expression. We therefore believe that HT of TEs could have an important impact on genome evolution.

TEs are highly dynamic sequences known to mutate rapidly. It is also commonly admitted that they can massively increase genome size through bursts of transpositional activity, as in the case of the wild rice species *O. australiensis *where three LTR-retrotransposons comprise 60% of the genome [11]. It is also well established that transposable elements are rapidly deleted from the genome [24,51]. These observations led some authors to propose the increase/decrease model for plant genome evolution [52] which postulates that bursts of transposition are rapidly counterbalanced by deletion. Based on the high rate of HTs involving *mariner-like *elements (MLES), it was further proposed that MLES could maintain themselves by horizontal transmission and thus counterbalance their elimination by stochastic loss and mutation [45,53]. Given the accumulation of the recent demonstrations of TE HTs, we propose to generalize this model of evolution to all classes of transposable elements. Horizontally transferred TEs can escape silencing and therefore transpose before they are deleted, increasing their chances of survival in their new host genome. HTs therefore allow TEs to colonize and invade new genomes by bursts of transposition as has been shown for *RIRE1 *in wild rice [9] or for the *P-element *in *Drosophila melanogaster *[7,15]. We propose that HTs may be a frequent process in the life cycle and evolution of TEs and that this phenomenon explains their persistence in the genomes of almost all eukaryotic genomes.

## Methods

### *In silico *analysis and identification of new candidate

We retrieved all rice retrotransposon sequences from the RetrOryza database ([54]). These were used in a Blastn search against available maize and *Sorghum *sequences. Conserved *Route66 *genomic copies of rice, maize and *Sorghum *were aligned using CLUSTALX [55] and modified by hand using SEAVIEW software [56]. The final alignment was used to construct trees using neighbour-joining methods with PAUP software [57]. 10,000 bootstrap replicates were performed.

### *Route66 *structural annotation

Annotation was performed using the Artemis software [58]. ORFs longer than 100 residues were automatically extracted from the element sequences. They were then scanned using a combination of Pfam, ProSite and Blastp analyses with standard parameters. The results were imported into Artemis in order to reconstruct the complete structure of each element. The LTRs were identified using Dotter [59] and the PBS and PPT were manually determined.

### Ka/Ks analysis and estimation of gene selective constraints

Both insertions of *Route66 *were mapped *in silico *on the genome of *O. sativa *(IRGSP pseudomolecules build 4) using RAPDB Blast . We retrieved each cDNA sequence with a flanking region spanning 1 Mb for each insertion (chromosomes 2 and 6). We found 78 and 115 cDNAs for chromosomes 2 and 6 respectively. These sequences were subsequently used as queries against the available sequences from the *Sorghum bicolor *genome. For the most significant Blastn hits, we used the phytozome web site  to retrieve sequences corresponding to *S. bicolor *mRNA. These mRNA sequences were then used for a Blastn search against the NCBI database in order to retrieve orthologous sequences from maize. Finally, maize sequences were used as queries against the NCBI database to check that they correspond to the initial rice transcript flanking the *Route66 *insertion. We selected 2 genes for each region (AK072088 and AB055156 for chromosome 2 and AK072921 and AK070134.1 for chromosome 6) with orthologous sequences in rice, *Sorghum *and maize. These sequences were aligned using the method described above. Sequence identities and non-synonymous to synonymous substitution ratios (Ka/Ks) were calculated using DNAsp software [60]. The same analyses were carried out for three genes: *Waxy *(granule bound starch synthase), *Nod26-like *(membrane protein) and *Gid1 *(Gibberellin receptor) which are not located in the flanking regions of *Route66 *but are known to be functional in the three species. Inter-specific sequence identities of coding region were thus computed for these seven selected genes.

We performed the same analysis with all putatively functional *Route66 *copies (with no stop codon in the coding region) of rice, maize and *Sorghum*, except that sequence identities were computed on the entire sequence of *Route66 *(4,890 pb) including both LTR and coding regions (Figure 2). The observed divergence was translated into an insertion date using a substitution rate of 1,3 × 10^-8 ^mutation/site/year [18].

### Amplification, cloning and phenetic analysis

Total DNA was extracted from 12 rice species provided by the International Rice Research Institute, Manila, Philippines: *Oryza. sativa*, *O. rufipogon *(10591), *O. longistaminata *(acc.110 404), *O. punctata *(acc.105 690), *O. officinalis *(acc.101 116), *O. minuta *(acc. 105 089), *O. alta *(acc.105 143), *O. australiensis *(acc. 100 882), *O. granulata *(102 118), *O brachyantha *(acc. 101 232), *O. ridleyi *(100821) and *O. coarctata *(acc. 104 502). DNA from *Panicum milliaceum *and from two bamboo species, *Phyllostachis bissetii *and *Phyllostachis aurea*, was also extracted. DNA from *Saccharum spontaneum, Saccharum officinarum *and *Saccharum robustum *and from *Sorghum bicolor, Sorghum aethiopicum*, *Sorghum arundinaceum*, *Sorghum drummondii*, *Sorghum propinquum *and *Sorghum virgatum *were provided by the Center for International Co-operation in Agronomic Research for Development (CIRAD, Montpellier, France). DNA of *Zea mays ssp mays *(maize), ssp *parviglumis *and ssp *mexicana *(teosinte) was provided by the station de génétique végétale (Le Moulon, Gif-sur-Yvette, France). DNA of *Triticum durum*, *Triticum monococum *and *Aegilops taushii *was provided by the ENSAM (Montpellier, France). DNA from *Brachypodium distachyon *was provided by the Faculty of Engineering and Natural Science Sabanci University Orhanli (Tuzla-Istanbul, Turkey) and DNA of wheat was provided by the INRA (Montpellier, France).

We used previous genomic sequence alignment of *Route66 *to design primers for its PCR amplification (Forward primer: 5'ACGCCGGAGTAGACCTCGTT3', Reverse primer: 5'ATATGCCATCTGTGGATATCC3'). The amplification products were cloned in pGEM-T easy vector (Promega, ). Sequences were obtained from both 5' and 3' ends to give a contig of around 1 kb. Only one representative sequence was kept per species and these were aligned with the corresponding regions of maize, rice and *Sorghum *genomic copies. Alignment and tree construction were performed as described above. Inter-species sequence identities were then calculated.

### *Adh1 *sequencing

In order to eliminate the possibility of DNA contamination, we amplified and sequenced *Adh1 *genes from *O. sativa*, *O. rufipogon*, *O. longistaminata*, *O. ridleyi*, in the three sugarcane species and in *Sorghum bicolor*. We used the primers designed by Ge et al. [61] to amplify the gene. For all the data obtained, we exclude that these results could be due to DNA contamination since we sequenced *Adh1 *gene for all the accessions we used in this study and confirmed that they correspond to the given species

## Abbreviations

TEs: Transposable Elements; HTs: Horizontal Transfers.

## Authors' contributions

AR carried out wet lab experiments, contributed to both *In silico *and phylogenetic analyses and to the writing of the manuscript. BP carried out the *in silico *analyses and contributed to the writing the manuscript. PMF performed the Ka/Ks and the phylogenetic analyses. FS performed the structural annotation. AD and DM contributed to biological sampling and data analysis. OP contributed to the writing of the manuscript and to the data analyses. All authors read and approved the final manuscript.

## Supplementary Material

Additional file 1 **Sequence identity between all genomic copies of *Route66*.** The data provided give the percentage of sequence identity between all genomic copies of *Route66 *identified in rice, *Sorghum *and maize. Click here for file

Additional file 2 **Alignment of all genomic copies of *Route66*.** The data provided give the alignment of all genomic copies of *Route66 *identified in rice, *Sorghum *and maize. Click here for file

Additional file 3 **Sequence identity between all 1 kb fragments.** The data provided give the percentage of sequence identity between all the 1 kb fragment of *Route66 *that we have sequenced. Click here for file

Additional file 4 **Ka/Ks supplemental values.** The data provided give the Ka/Ks values calculated between all genomic copies of *Route66 *identified in rice, *Sorghum *and maize.
Click here for file
