# Supplementary material for: Whole genome surveys of rice, maize and sorghum reveal multiple horizontal transfers of the LTR-retrotransposon Route66 in Poaceae
Source: BMC Evol Biol. 2009 Mar 16;9:58. doi: 10.1186/1471-2148-9-58 (PMC2664808; doi:10.1186/1471-2148-9-58)
Supplement: Additional file 1 — Sequence identity between all genomic copies of Route66. The data provided give the percentage of sequence identity between all genomic copies of Route66 identified in rice, Sorghum and maize. [file 1471-2148-9-58-S1.pdf]

	sb2	sb4	sb3	sb5	zm2	zm3	zm1	osj1	osj2	osi1	sb1	zm4	zm5	zm7	zm6	zm9	zm11	zm12	zm10	zm8
sb2																				
sb4	98,3																			
sb3	98,3	98,3																		
sb5	95,1	95,1	94,9																	
zm2	89,1	89,3	89,2	87,1																
zm3	90,5	90,8	90,7	88,3	93,8															
zm1	91,2	91,4	91,3	89,0	93,5	95,2														
osj1	89,4	89,2	89,4	86,6	87,2	88,6	89,2													
osj2	89,2	89,1	89,3	86,5	87,0	88,5	89,0	99,4												
osi1	89,0	89,0	89,1	86,3	87,0	88,4	88,9	99,1	99,5											
sb1	87,7	87,7	87,9	85,4	86,5	87,7	88,1	95,4	95,3	95,2										
zm4	87,5	87,3	87,7	85,0	85,6	86,6	87,2	92,0	91,9	91,8	90,8									
zm5	87,5	87,3	87,7	85,0	85,6	86,6	87,2	92,0	91,9	91,8	90,8	100,0								
zm7	87,4	87,1	87,5	84,6	85,2	86,5	87,0	91,5	91,3	91,2	89,9	96,9	96,9							
zm6	88,0	87,7	88,0	85,4	86,0	86,9	87,6	92,3	92,1	92,0	90,9	97,8	97,8	98,4						
zm9	88,1	87,8	88,2	85,5	85,9	87,1	87,9	92,4	92,3	92,2	91,0	97,9	97,9	98,6	99,1					
zm11	88,1	87,8	88,1	85,4	85,9	87,0	87,7	92,4	92,3	92,1	91,0	97,9	97,9	98,3	99,0	99,4				
zm12	88,1	87,8	88,1	85,4	85,9	87,0	87,7	92,4	92,3	92,1	91,0	97,9	97,9	98,3	99,0	99,4	100,0			
zm10	88,2	87,8	88,2	85,5	86,0	87,1	87,9	92,4	92,3	92,1	91,1	97,9	97,9	98,6	99,1	99,5	99,5	99,5		
zm8	88,2	87,9	88,2	85,6	86,0	87,1	87,7	92,5	92,4	92,2	91,0	97,8	97,8	98,7	99,1	99,3	99,1	99,1	99,2	
