# Supplementary material for: Whole genome surveys of rice, maize and sorghum reveal multiple horizontal transfers of the LTR-retrotransposon Route66 in Poaceae
Source: BMC Evol Biol. 2009 Mar 16;9:58. doi: 10.1186/1471-2148-9-58 (PMC2664808; doi:10.1186/1471-2148-9-58)
Supplement: Additional file 3 — Sequence identity between all 1 kb fragments. The data provided give the percentage of sequence identity between all the 1 kb fragment of Route66 that we have sequenced. [file 1471-2148-9-58-S3.pdf]

	sb2	sb4	sb3	sb5	zm2	zm3	zm1	osj1	osj2	osi1	sb1	zm4	zm5	zm7	zm6	zm9	zm11	zm12	zm10	zm8	O. rufi	O. lo	O.rid	zmm	zmp	sd	sae	sa	sv	pa	pb	sp	sr	soff	ssp
sb2																																			
sb4	100,0																																		
sb3	100,0	98,0																																	
sb5	96,3	96,4	95,3																																
zm2	89,0	89,0	88,6	88,2																															
zm3	89,7	89,7	89,2	88,4	94,9																														
zm1	89,8	89,8	89,3	88,0	94,1	95,4																													
osj1	86,5	86,7	86,0	84,6	87,1	88,6	88,5																												
osj2	86,9	87,0	86,4	85,0	87,0	88,6	88,5	99,4																											
osi1	86,9	87,0	86,4	85,0	87,0	88,6	88,5	99,4	100,0																										
sb1	85,7	85,9	85,4	84,2	86,7	88,3	89,0	95,2	95,3	95,3																									
zm4	85,0	85,1	84,4	83,0	84,3	85,4	85,7	90,1	90,5	90,5	89,9																								
zm5	85,0	85,1	84,4	83,0	84,3	85,4	85,7	90,1	90,5	90,5	89,9	100,0																							
zm7	84,6	84,7	83,9	82,4	83,8	85,0	85,3	90,0	90,4	90,4	89,7	97,3	97,3																						
zm6	85,7	85,8	85,0	83,2	84,8	85,9	86,2	91,0	91,4	91,4	90,8	97,8	97,8	98,5																					
zm9	86,0	86,1	85,2	83,5	84,9	86,3	86,6	91,1	91,5	91,5	90,8	98,0	98,0	98,8	99,3																				
zm11	85,8	86,0	85,1	83,4	84,8	86,2	86,5	91,3	91,7	91,7	90,9	97,9	97,9	98,4	99,0	99,4																			
zm12	85,8	86,0	85,1	83,4	84,8	86,2	86,5	91,3	91,7	91,7	90,9	97,9	97,9	98,4	99,0	99,4	100,0																		
zm10	85,8	86,0	85,1	83,4	84,8	86,2	86,5	91,3	91,7	91,7	90,9	98,0	98,0	98,6	99,2	99,7	99,7	99,7																	
zm8	85,5	85,7	85,0	83,2	84,4	85,8	86,1	90,9	91,3	91,3	90,5	97,9	97,9	98,9	99,2	99,4	99,1	99,1	99,3																
O.rufi	85,6	85,7	84,8	83,4	85,3	87,3	86,6	97,0	97,6	97,6	93,2	88,5	88,5	88,7	89,4	89,6	89,7	89,7	89,7	89,3															
O.longi	86,8	86,9	86,2	84,8	87,1	88,6	88,5	98,6	98,7	98,7	95,6	90,4	90,4	90,4	91,4	91,5	91,6	91,6	91,6	91,2	96,6														
O. rid	84,8	85,0	84,2	83,3	84,9	86,7	86,6	95,3	95,4	95,4	92,9	88,8	88,8	88,5	89,3	89,6	89,7	89,7	89,7	89,3	93,4	95,8													
zmm	85,5	85,6	84,8	83,1	84,3	85,7	86,0	90,1	90,5	90,5	89,8	97,3	97,3	98,1	98,6	99,3	98,9	98,9	99,0	98,7	88,6	90,5	88,5												
zmp	85,5	85,6	84,8	83,2	84,9	86,1	86,4	91,0	91,4	91,4	90,8	97,8	97,8	98,5	99,0	99,4	99,7	99,7	99,7	99,1	89,4	91,4	89,3	98,8											
sd	84,5	84,6	83,9	83,7	86,0	88,3	88,3	95,2	95,3	95,3	99,7	89,5	89,5	89,4	90,0	90,0	90,4	90,4	90,2	89,6	92,2	95,7	91,8	89,1	89,9										
sae	84,0	84,0	83,4	83,2	85,7	88,0	88,0	95,0	95,2	95,2	99,5	89,3	89,3	89,2	89,8	89,8	90,2	90,2	90,0	89,4	92,0	95,6	92,2	88,9	89,7	99,5									
sae	85,3	85,4	85,3	83,8	86,4	88,2	87,8	94,7	94,9	94,9	96,6	88,6	88,6	88,5	89,4	89,4	89,5	89,5	89,5	89,3	92,6	95,1	92,5	88,2	89,2	95,9	95,7								
sv	85,9	86,1	85,6	84,6	86,8	88,2	88,8	95,1	95,2	95,2	99,6	89,9	89,9	89,7	90,8	90,8	90,9	90,9	90,9	90,5	93,1	95,5	92,8	89,6	90,6	99,7	99,5	96,5							
pa	89,2	89,0	88,7	86,9	89,0	91,2	90,9	92,1	92,2	92,2	92,2	88,3	88,3	88,1	89,0	89,3	89,3	89,3	89,3	88,9	90,9	92,3	90,2	88,3	88,9	91,5	91,4	91,2	92,0						
pb	89,4	89,1	88,7	87,0	89,0	91,2	90,9	92,2	92,3	92,3	92,4	88,5	88,5	88,2	89,2	89,4	89,4	89,4	89,4	89,0	90,9	92,4	90,2	88,4	89,0	91,5	91,3	91,3	92,1	99,8					
sp	84,8	84,9	84,7	83,3	86,0	87,9	87,5	94,5	94,6	94,6	95,3	88,2	88,2	88,0	88,9	88,9	89,0	89,0	89,0	88,7	92,4	94,9	92,5	87,8	88,7	95,3	95,1	97,8	95,2	91,0	91,0				
sr	84,9	85,0	84,4	83,4	85,1	86,5	86,3	94,4	94,6	94,6	93,1	87,7	87,7	87,8	88,4	88,7	88,8	88,8	88,8	88,4	92,7	94,9	93,1	87,7	88,4	92,2	91,9	93,7	93,0	90,9	90,9	93,8			
soff	85,6	85,7	85,2	83,8	85,6	87,1	86,8	95,5	95,5	95,5	94,1	89,0	89,0	88,8	89,6	89,9	90,0	90,0	90,0	89,6	94,0	96,0	94,1	88,9	89,8	92,7	92,5	94,8	94,0	92,0	92,1	94,9	97,7		
ssp	84,6	84,7	84,2	82,6	84,5	85,7	85,8	94,0	94,0	94,0	92,9	88,2	88,2	88,1	88,6	88,9	89,0	89,0	89,0	88,9	92,2	94,4	92,7	87,8	88,6	91,7	91,5	93,0	93,1	90,2	90,2	93,2	95,6	97,1	
